# Auranofin Rapidly Eradicates Methicillin-resistant *Staphylococcus aureus* (MRSA) in an Infected Pressure Ulcer Mouse Model

**DOI:** 10.1038/s41598-020-64352-2

**Published:** 2020-04-29

**Authors:** Haroon Mohammad, Nader S. Abutaleb, Mohamed N. Seleem

**Affiliations:** 1 Department of Comparative Pathobiology, College of Veterinary Medicine, Purdue University, 625 Harrison St., West Lafayette, IN 47907 USA; 2 Purdue Institute for Inflammation, Immunology, and Infectious Disease, 610 Purdue Mall, West Lafayette, IN 47907 USA

**Keywords:** Drug discovery, Skin models, Bacteria

## Abstract

Pressure ulcers (PUs) frequently occur in individuals with limited mobility including patients that are hospitalized or obese. PUs are challenging to resolve when infected by antibiotic-resistant bacteria, particularly methicillin-resistant *Staphylococcus aureus* (MRSA). In this study, we investigated the potential of repurposing auranofin to treat pressure ulcers infected with MRSA. Auranofin’s *in vitro* activity against strains of *S. aureus* (including MRSA) was not affected in the presence of higher bacterial inoculum (10^7^ CFU/mL) or by lowering the pH in standard media to simulate the environment present on the surface of the skin. Additionally, *S. aureus* did not develop resistance to auranofin after repeated exposure for two weeks via a multi-step resistance selection experiment. In contrast, *S. aureus* resistance to mupirocin emerged rapidly. Moreover, auranofin exhibited a long postantibiotic effect (PAE) *in vitro* against three strains of *S. aureus* tested. Remarkably, topical auranofin completely eradicated MRSA (8-log_10_ reduction) in infected PUs of obese mice after just four days of treatment. This was superior to both topical mupirocin (1.96-log_10_ reduction) and oral clindamycin (1.24-log_10_ reduction), which are used to treat infected PUs clinically. The present study highlights auranofin’s potential to be investigated further as a treatment for mild-to-moderate PUs infected with *S. aureus*.

## Introduction

Pressure ulcers (PUs), also commonly referred to as pressure sores or decubitus ulcers, are defined as “localized injury to the skin and/or underlying tissue” that often form when skin is compressed between bone and an external surface^1^. In the United States (U.S.) alone, nearly 1.6 million pressure ulcers develop in hospitalized patients each year^2^. Additionally, PUs are the second leading source of hospital readmissions each year, impacting between 1.3 and 3 million people^3^.

Bacterial infection of PUs is common and problematic as infection can (1) impair healing of the ulcerated tissue, (2) lead to more serious disseminated infections including osteomyelitis and sepsis, and (3) result in increased treatment costs^4–6^. *Staphylococcus aureus* and Gram-negative bacilli are the most frequent bacterial pathogens linked to infected PUs^4,7^. Such infections can be treated with systemic (such as clindamycin) or topical antibiotics (such as mupirocin or fusidic acid)^7,8^. However, the formation of biofilms within PUs that protect the pathogen from the effect of many antibiotics, secretion of toxins by the pathogen that lead to further damage of the skin and surrounding tissues, and the emergence of multidrug-resistant strains, including methicillin-resistant *S. aureus* (MRSA) and vancomycin-resistant *S. aureus* (VRSA), have compounded treatment of infected PUs^4^. Thus, identifying new antibacterial agents effective in treating PUs infected with antibiotic-resistant bacteria are needed.

Auranofin is a gold-containing drug that received FDA approval in 1985 as a second-line therapeutic to treat rheumatoid arthritis. Recently, there has been renewed interest in repurposing auranofin to treat an array of medical diseases including cancer, parasitic infections, and bacterial skin and soft tissue infections^9–14^. As infected PUs are a notable challenge to obese individuals, the main objective of the present study was to investigate auranofin’s ability to reduce the burden of MRSA in infected PUs in obese mice.

## Results

### Antibacterial activity of auranofin against staphylococcal isolates under acidic pH and high inoculum conditions

Previous reports have investigated the *in vitro* antibacterial activity of auranofin in standard media (such as cation-adjusted Mueller-Hinton broth or Tryptic soy broth at neutral pH) with a fixed bacterial inoculum size (~10^5^ CFU/mL), following guidelines provided by the Clinical and Laboratory Standards Institute. Under these standard conditions, we determined that auranofin exhibited potent *in vitro* antibacterial activity against strains of methicillin-sensitive *S. aureus*, MRSA, and VRSA with minimum inhibitory concentration (MIC) values ranging from 0.015 –0.06 µg/mL (Table 1). These results are in agreement with previous reports^13–15^. The activity of auranofin was similar to clindamycin (MIC ranged from 0.015–0.03 µg/mL), although clindamycin was ineffective against the two VRSA strains tested (MIC > 8 µg/mL). Auranofin exhibited more potent *in vitro* activity against all six clinical staphylococcal isolates tested relative to mupirocin (MIC ranged from 0.06–0.25 µg/mL, except for clinical isolate *S. aureus* NRS107 which exhibits high-level resistance to mupirocin).

**Table 1:** Minimum inhibitory concentration (MIC, in µg/mL) of auranofin and control antibiotics (clindamycin and mupirocin) tested against strains of *Staphylococcus aureus* in the presence of different inoculum size and acidic pH.

Bacterial Strain	Genotype	Auranofin				Clindamycin				Mupirocin			
		Inoculum size (CFU/mL)			pH 6.0	Inoculum size (CFU/mL)			pH 6.0	Inoculum size (CFU/mL)			pH 6.0
		10^5^	10^6^	10^7^		10^5^	10^6^	10^7^		10^5^	10^6^	10^7^	
ATCC 6538^a^	*mecA*^−^	0.03	0.03	0.06	0.03	0.03	0.06	0.125	0.25	0.06	0.125	0.125	0.0039
NRS107^a^	*mecA*^−^	0.015	0.015	0.03	0.0039	0.015	0.015	0.03	0.125	>8	>8	>8	>8
NRS384 (USA300)^b^	*mecA*^+^ (subtype IV); *pvl*^+^	0.03	0.03	0.06	0.03	0.03	0.06	0.25	0.50	0.25	0.25	0.50	0.015
NRS123 (USA400)^b^	*mecA*^+^ (subtype IV); *pvl*^+^; *seb*^+^	0.06	0.06	0.06	0.06	0.03	0.03	0.25	0.50	0.25	0.25	0.50	0.015
VRS9^c^	*mecA*^+^; *vanA*^+^	0.06	0.06	0.06	0.03	>8	>8	>8	>8	0.125	0.125	0.25	0.0078
VRS12^c^	—	0.06	0.06	0.06	0.03	>8	>8	>8	>8	0.25	0.25	0.50	0.0078

^a^Methicillin-sensitive *S. aureus*.

^b^Methicillin-resistant *S. aureus* (MRSA).

^c^Vancomycin-resistant *S. aureus* (VRSA).

We next evaluated the impact of bacterial inoculum size on the antibacterial activity of auranofin. Auranofin’s antibacterial activity was identical to or two-fold higher as the inoculum size increased from 10^5^ CFU/mL to 10^6^ CFU/mL to 10^7^ CFU/mL (Table 1), suggesting the drug’s activity was not impacted by inoculum size. This was similar to the result observed with mupirocin, in agreement with a previous study^16^. In contrast, clindamycin’s activity was negatively affected as the bacterial inoculum size increased (MIC increased two- to eight-fold against *S. aureus* ATCC 6538, *S. aureus* NRS107, MRSA NRS123, and MRSA NRS384).

In addition to examining the impact of bacterial inoculum size on the antibacterial activity of auranofin, we also investigated the impact of changing the pH of the media. Auranofin’s antibacterial activity was similar to or slightly more potent in media that was acidic (pH 6.0), MIC was two- to four-fold lower against *S. aureus* NRS107 and VRS9, compared to media at neutral pH (Table 1), suggesting that the drug’s activity should remain stable or be slightly enhanced in acidic environments, such as the surface of the skin. Both clindamycin’s and mupirocin’s antibacterial activity were affected by pH. Under acidic pH conditions, clindamycin’s MIC was negatively impacted and increased eight- to 16-fold relative to the antibiotic’s activity at neutral pH. In contrast, mupirocin’s antibacterial activity was enhanced by 16- to 32-fold (MIC ranged from 0.0039–0.015 µg/mL). The enhanced activity of mupirocin under acidic conditions is known and is advantageous for topical treatment of *S. aureus* skin and wound infections^16,17^.

### Evaluation of *S. aureus* resistance formation to auranofin after multiple exposures

**Figure 1:**
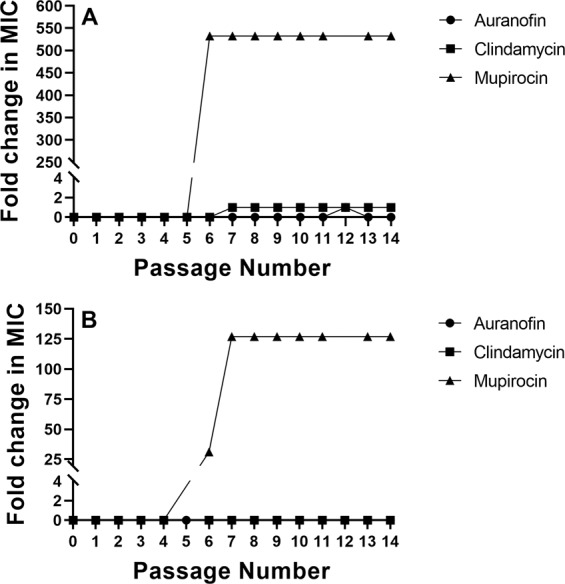
Multi-step resistance selection for auranofin, clindamycin, and mupirocin evaluated against *S. aureus*. Drugs were serially passaged daily against (**A**) *S. aureus* ATCC 6538 and (**B**) methicillin-resistant *S. aureus* (MRSA) NRS384 (USA300) for 14 days. The minimum inhibitory concentration (MIC) of each test agent was determined after each passage. Data are presented as fold-change in MIC relative to the previous passage.

We moved to evaluate the ability of *S. aureus* to develop resistance to auranofin after multiple exposures to the drug, similar to how the drug would be administered clinically to resolve a bacterial infection. A multi-step resistance selection experiment with auranofin was conducted with two strains of *S. aureus* (*S. aureus* ATCC 6538 and MRSA NRS384) to investigate this issue further. No shift in the MIC values for auranofin against either *S. aureus* strain was observed during the duration of the study (Fig. 1). Similar to auranofin, neither strain of *S. aureus* developed resistance to clindamycin over the 14-day period. Only a one-fold increase in the MIC of clindamycin was observed after the seventh passage against *S. aureus* ATCC 6538. In contrast, *S. aureus* ATCC 6538 developed resistance to mupirocin after the fifth passage (MIC increased >500-fold) while MRSA NRS384 developed resistance to mupirocin after the fourth passage (MIC increased >30-fold). This was similar to previous studies that found clinical isolates of *S. aureus* developed resistance to mupirocin rapidly after 2 to 14 days of exposure via a multi-step resistance selection study^18,19^.

### Postantibiotic effect of auranofin and control antibiotics against *S. aureus* isolates

Auranofin exhibited a long PAE of seven hours against two MRSA strains (NRS123 and NRS384) (Table 2). Against *S. aureus* ATCC 6538, the PAE of auranofin exceeded nine hours. Clindamycin exhibited a PAE that ranged from four hours (against MRSA NRS123) to six hours (against *S. aureus* ATCC 6538). The variation in PAE for clindamycin is in agreement with Xue *et al*.’s report, that found the PAE of clindamycin against 21 strains of *S. aureus* ranged from 0.4 to 3.9 hours and was dependent on length of exposure and concentration of drug used^20^. Mupirocin exhibited a shorter PAE of four hours, under the same conditions, against all three strains of *S. aureus*. This was similar to a previous study by Rittenhouse *et al*., that found mupirocin (at 4 × MIC) exhibited a short PAE that ranged between 2.2 and 2.9 hours^21^.

**Table 2:** Postantibiotic effect of auranofin and control antibiotics (clindamycin and mupirocin) tested (at 5 × MIC) against strains of *Staphylococcus aureus*.

Bacterial Strain	Postantibiotic Effect (hours)		
	Auranofin	Clindamycin	Mupirocin
*S. aureus* ATCC 6538	>9	6	4
MRSA NRS384 (USA300)	7	5	4
MRSA NRS123 (USA400)	7	4	4

### Auranofin eradicates MRSA in infected pressure ulcers *in vivo* in obese mice

**Figure 2:**
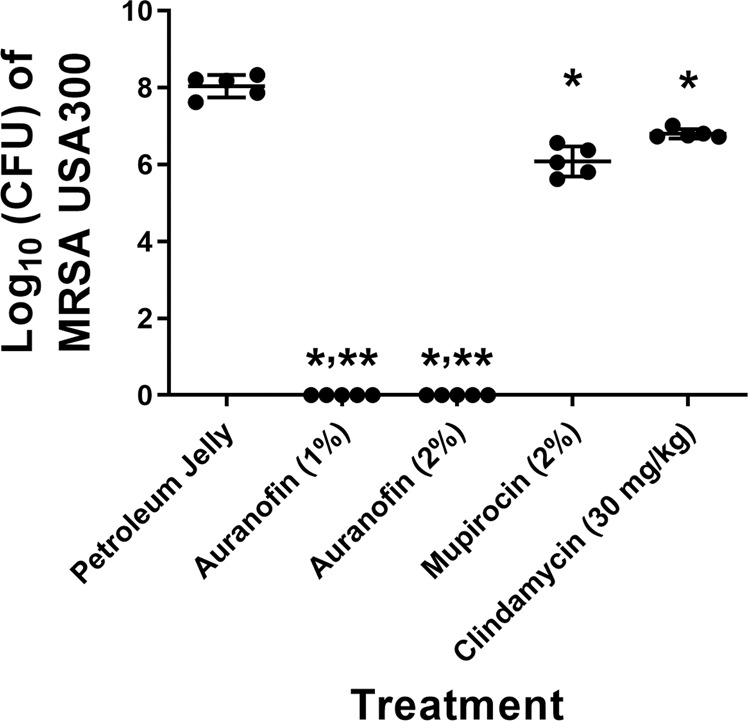
Burden of methicillin-resistant *S. aureus* (MRSA) USA300 in the wounds of obese mice after treatment with auranofin and control antibiotics. The dorsum of female TALLYHO/JngJ were exposed to 10 cycles (two hours on, one hour off) of rare earth magnets to induce the formation of pressure ulcers. Ulcers were infected with MRSA USA300 and 48 hours post-infection were treated topically either with auranofin (1% or 2%) or mupirocin (2%) twice daily for four days (n = 5 mice/group). One group of mice received oral clindamycin (30 mg/kg) once daily and another group received the vehicle alone (petroleum jelly administered topically) twice daily for four days. Mice were humanely euthanized 12 hours after the final treatment dose and wounds were harvested aseptically to determine reduction in bacterial burden post-treatment. Data are presented as log_10_ (total MRSA CFU per wound) for each mouse and were evaluated using a one-way ANOVA with post-hoc Dunnet’s test for multiple comparisons. One asterisk (*) indicates statistical difference for test agents relative to petroleum jelly (negative control, *P* < 0.05). Two asterisks (**) indicates statistical different between auranofin and mupirocin-treated mice (*P* < 0.05).

Auranofin was superior (*P* < 0.0001) to both topical mupirocin and oral clindamycin in reducing the burden of MRSA in infected PUs of obese TALLYHO/JngJ mice. After just four days of treatment, auranofin (2% topical suspension) completely eradicated MRSA from the infected PUs in all five mice (Fig. 2). This was equivalent to reducing the bacterial burden by 8-log_10_ relative to mice receiving the negative control (vehicle alone). Reducing the concentration of auranofin to a 1% topical preparation yielded the same result as no viable MRSA colonies were present in infected PUs of all five mice. Topical mupirocin reduced the burden of MRSA by 1.96-log_10_ (*P* < 0.0001) in infected PUs. Oral clindamycin, though effective in producing a statistically significant reduction in bacterial burden, was the least potent antibacterial as clindamycin reduced the burden of MRSA in infected PUs by only 1.24-log_10_ (*P* < 0.0001).

## Discussion

Pressure ulcers significantly impact individuals that are immobilized or exhibit limited mobility, including patients in hospitals and individuals that are obese. Obesity is often linked to other co-morbidities, including hypertension and Type 2 diabetes, which can result in hospitalizations. Indeed, 25% of patients that present in hospital intensive care units (ICU) are obese^22^. The rate of obesity has tripled in the past 40 years and impacts nearly 13% of the adult population (>650 million people as of 2016) worldwide and nearly 40% of the adult population in the U.S. (>93 million individuals)^23,24^. Due to limited mobility, obese individuals, both in the ICU and in the community setting, tend to be at a higher risk of developing pressure ulcers^22^. Bacterial infections of pressure ulcers further complicate treatment and healing of PUs. Increased bacterial burden in PUs impedes the formation of granulation tissue which can delay wound healing^6^. It has been postulated that healing of PUs is hindered when the bacterial population exceeds 10^5^ CFU/g, though more recent studies have found that bacterial populations below the 10^5^ CFU/g threshold may be deleterious to healing of infected PUs^6,25^. Thus, finding antibacterial agents capable of rapidly eliminating bacteria in infected PUs would potentially aid in enhancing resolution of infected PUs. Antibiotics currently used to treat PUs infected with bacteria, such as *S. aureus*, may be ineffective due to multiple factors including bacterial resistance to the antibiotic, the presence of biofilms, and inability to neutralize toxins secreted by bacteria that further damage the infected lesions. Identifying novel therapeutics capable of treating infected PUs are needed.

As noted in the introduction, auranofin was originally approved for the treatment of rheumatoid arthritis. Though auranofin had the advantage of being an orally-administered gold drug and was associated with fewer adverse reactions in patients, injectable gold compounds were found to be more effective in resolving symptoms associated with rheumatoid arthritis^26,27^. This reason combined with the emergence of more potent antirheumatic agents, such as oral methotrexate, resulted in decreased clinical use of auranofin by the early 1990s^28^. However, recent studies have investigated repurposing auranofin as an antibacterial agent. Auranofin has potent activity against important Gram-positive bacterial pathogens including MRSA and vancomycin-resistant *Staphylococcus aureus* (VRSA) and exerts its antibacterial effect by inhibiting multiple biosynthetic pathways, including protein synthesis^13^. Previously, we demonstrated that auranofin applied topically is effective in significantly reducing the burden of MRSA in an uncomplicated abscess model in mice and in decreasing expression of pro-inflammatory cytokines (TNF-α, MCP-1, IL-1β, and IL-6) that may impair wound healing^14^. Furthermore, auranofin inhibited the production of key toxins (Panton-Valentine leucocidin and α-hemolysin) produced by MRSA that damage host tissues and was effective in eradicating *S. aureus* biofilm *in vitro*^13,14^. These features, we hypothesized, would be beneficial in treating mild-to-moderate pressure ulcers infected with MRSA.

Initially, we investigated the effect of increasing the bacterial inoculum size and the effect of pH on the antibacterial activity of auranofin. Though an inoculum size ~10^5^ CFU/mL is used in standard antibacterial susceptibility assays, a higher inoculum (>10^6^ CFU/mL) is often used to infect animals in *in vivo* infection models. Additionally, infected PUs clinically present with a bacterial burden that exceeds 10^5^ CFU/g^6^. We thus evaluated the impact of increasing the bacterial inoculum (above standard broth microdilution assay conditions) on the antibacterial activity of auranofin. Auranofin’s *in vitro* antibacterial activity increased two-fold against three of the six *S. aureus* strains tested as the bacterial inoculum size increased from 10^5^ to 10^7^ CFU/mL. Next, we assessed the effect of pH on auranofin’s antibacterial activity. The skin surface represents an acidic environment (pH ranging from 4.0 to 6.0) which differs from the neutral pH used in standard media^29^. Thus, to determine if the acidic environment on the surface of the skin may affect auranofin’s antibacterial activity, particularly if used topically, the MIC of auranofin in acidic conditions (media adjusted to pH 6.0) was determined. Auranofin’s antibacterial activity was unaffected or slightly more potent under acidic conditions (pH 6.0), compared to media at neutral pH. This indicates that auranofin’s activity may be slightly enhanced in acidic environments, such as the surface of the skin.

After evaluating the effect of bacterial inoculum size and pH on the antibacterial activity of auranofin, we next evaluated the ability of *S. aureus* to develop resistance to auranofin. Bacteria have a proclivity to acquire or develop resistance to antibiotics, particularly after repeated exposure. Previously, our research group determined that *S. aureus* was unable to develop spontaneous resistance to auranofin (at 3 ×, 5 ×, and 10 × MIC) via a single-step resistance selection experiment^13^. Harbut *et al*., also noted the inability to isolate *Mycobacterium tuberculosis* spontaneous mutants exhibiting resistance to auranofin^11^. Using a multi-step resistance selection assay, both *S. aureus* ATCC 6538 and MRSA NRS384 did not develop resistance to auranofin (no shift in the MIC) after 14 passages. This was similar to a recent report investigating auranofin’s activity against vancomycin-resistant *Enterococcus faecium*^30^, which indicates bacteria are unlikely to develop resistance rapidly to auranofin.

After determining that the antibacterial activity of auranofin was not impacted by a lower pH or higher bacterial inoculum and confirming the low potential of *S. aureus* to develop resistance to auranofin after multiple passages, we moved to investigate if auranofin exhibits a postantibiotic effect (PAE). In the 1940s, Parker *et al*., noted that penicillin could suppress growth of staphylococci, even after a very short exposure to the antibiotic^31^. This phenomenon was later termed as postantibiotic effect and has been proposed to impact dosing regimens for antibiotics. Antibiotics that exhibit a long PAE are thought to be beneficial, as it is postulated that these agents may require fewer doses clinically^20^. Auranofin (at 5 × MIC) exhibited a long PAE that ranged from seven hours against two MRSA strains to over nine hours against *S. aureus* ATCC 6538. These results suggest auranofin is capable of suppressing *S. aureus* growth for a long period of time, even after a short exposure to the drug. This would potentially reduce the frequency that auranofin would need to be administered clinically to treat an infection caused by *S. aureus*.

The final step in our study was to evaluate auranofin’s ability to reduce the burden of *S. aureus* in infected PUs in an animal model. In order to evaluate the effectiveness of auranofin in treating infected PUs, we developed a model using female TALLYHO/JngJ mice. Female TALLYHO/JngJ mice represent a new animal model to investigate different disease indications in obese mice that are nondiabetic^32^. This particular strain of mice exhibits characteristic features of obesity including increased weight gain, moderate hyperleptinemia, moderate hyperinsulinemia, as well as impaired wound healing^33,34^. Utilizing two neodymium rare Earth magnets applied via a series of ischemia-reperfusion cycles, moderate ulcers that exhibited full-thickness skin loss with limited exudate were formed. The magnets possessed a strength of 3,466 G which exceeds 50 mm Hg compression pressure. This is important as a previous study found a compression pressure that exceeds 50 mm Hg will decrease capillary blood flow by 80% resulting in decreased oxygen delivery to cells, ultimately resulting in cell death^35^. The pressure ulcers were subsequently infected with MRSA NRS384 before treatment was initiated. Topical auranofin (both at 1% and 2%) rapidly eradicated the burden of MRSA in the infected wounds after only four days of treatment. This was superior to both topical mupirocin (2%) and oral clindamycin (30 mg/kg).

Though clindamycin is used clinically to treat skin infections and wounds caused by *S. aureus*, several issues have been noted. First, clindamycin usage has been linked to gastrointestinal toxicity and diarrhea^36^. Second, clindamycin’s antibacterial activity against Gram-positive bacteria and anaerobic Gram-negative rods can lead to dysbiosis of the natural microflora present in the gastrointestinal tract and increase susceptibility to infections by opportunistic pathogens, such as *Clostridioides difficile*^36^. Identifying alternative options to the use of systemic antibacterial agents, including the use of topical antibacterials capable of rapidly eliminating bacteria in infected PUs such as auranofin, would be beneficial. However, it should be noted that the most recent guidelines from the European Pressure Ulcer Advisory Panel (EPUAP), National Pressure Injury Advisory Panel (NPIAP) and Pan Pacific Pressure Injury Alliance (PPPIA) on the treatment of infected pressure ulcers only recommends the use of systemic antibiotics^37^. However, topical antiseptics, including those active against biofilms, are recommended to use to control bacterial burden in infected PUs that exhibit delayed wound healing. Though use of topical antibiotics is not included in the current treatment guidelines for infected PUs, the use of a topical antibiotic to treat wounds infected with bacteria has been proposed to have multiple benefits including delivering a high concentration of drug directly to the site of infection, decreasing the likelihood of systemic toxicity to the host, and easier/better patient compliance^38^.

The positive result observed with auranofin in our infected PU mice model was more pronounced compared to previous studies that have investigated auranofin as a topical antibacterial agent to treat MRSA skin wound infections^14,39^. For example, Thangamani *et al*., found that auranofin applied topically to uncomplicated skin abscesses in mice resulted in a 2.51-log_10_ reduction (for 1% auranofin) to 3.64-log_10_ reduction (for 2% auranofin) in MRSA burden^14^. We suspect the differences observed between these mice studies are due to multiple factors including 1.) different species of mice used (for example healthy BALB/c or CD-1 mice compared to TALLYHO/JngJ obese mice in our study), 2.) how skin wounds were formed (intradermal injection of bacteria to induce formation of simple abscesses compared to magnets to induce pressure ulcer formation in our study), and 3.) the use of a wound dressing (Tegaderm) in our study to hinder mice from grooming/removing drug from the infection site which permitted enhanced contact time for the drug to exert its effect. Wound dressings such as Tegaderm have an additional benefit in that they are permeable to water vapor and oxygen, which helps keep wounds moist, and prevents contamination of wounds by other microorganisms^40^. Previous studies have found that wounds that are kept moist heal more rapidly as an optimal environment is present for cells involved in wound healing^41–44^. Thus, the incorporation of a wound dressing into our PU mouse model, we suspect, played a role in the enhanced efficacy of auranofin observed relative to other published reports.

In conclusion, pressure ulcers are a common occurrence in individuals with limited mobility including patients that are hospitalized or obese. In this study, we investigated the effectiveness of auranofin, as a new antibacterial agent, to treat pressure ulcers infected with MRSA. Auranofin’s antibacterial activity *in vitro* was stable even in the presence of acidic pH or high bacterial inoculum size compared to both clindamycin and mupirocin. In obese mice, topical auranofin was superior to both topical mupirocin and oral clindamycin in rapidly eliminating the burden of MRSA in infected PUs. The complete eradication of MRSA from the PUs is postulated to be beneficial to aid in wound repair and healing though further studies will be needed to investigate this point in more depth.

## Methods

### Antibiotics and reagents

Staphylococcal clinical isolates were acquired from the American Type Culture Collection (ATCC, Manassas, VA, USA) or the Biodefense and Emerging Infections Research Resources Repository (BEI Resources, Manassas, VA, USA). Auranofin (Chem-Impex International, Wood Dale, IL, USA), clindamycin hydrochloride monohydrate (Tokyo Chemical Industry Co., Tokyo, Japan), and mupirocin (PanReac AppliChem ITW Reagents, Darmstadt, Germany) were purchased from commercial vendors and dissolved either in sterile water or in dimethyl sulfoxide (DMSO) to prepare stock 10 mg/mL solutions. Cation-adjusted Mueller Hinton broth (CA-MHB, Becton, Dickinson and Company, Sparks, MD, USA), Tryptic soy agar (TSA, Hardy Diagnostics, Santa Maria, CA, USA), Tryptic soy broth (TSB, Becton, Dickinson and Company, Sparks, MD, USA), mannitol salt agar (Hardy Diagnostics, Santa Maria, CA, USA), phosphate-buffered saline (PBS, Corning, Manassas, VA, USA), hydrochloric acid (HCl, Fisher Scientific, Fair Lawn, NJ, USA), petroleum jelly, rare earth magnets (Magcraft, Vienna, VA, USA), betadine solution (Fisher Scientific, Fair Lawn, NJ, USA), Tegaderm (3 M, St. Paul, MN, USA), Uro-bond V (Urocare, Pomona, CA, USA), and 96-well plates were all purchased from commercial vendors. Buprenorphine was provided by the Purdue Translational Pharmacology Core at Purdue University.

### Evaluation of the effect of bacterial inoculum size and pH on auranofin and control antibiotics’ antibacterial activity

The broth microdilution assay was used to determine the minimum inhibitory concentration (MIC) of auranofin, clindamycin, and mupirocin against six different clinical isolates of *S. aureus*^45^. A preparation of *S. aureus* to a McFarland 0.5, in sterile PBS, was subsequently diluted 1:3 (to reach 10^7^ CFU/mL), 1:30 (to reach 10^6^ CFU/mL), or 1:300 (to reach 10^5^ CFU/mL) in sterile CA-MHB (pH 7.4). An aliquot of each inoculum preparation was serially-diluted and plated onto TSA to confirm the initial inoculum size. To investigate the effect of pH on antibacterial activity, an aliquot of 1 M HCl was added to CA-MHB until a pH of 6.0 ± 0.1 was reached. Test agents were added in triplicate wells to a 96-well plate and serially diluted two-fold with the bacterial inoculum. Plates were incubated at 37°C for at least 18 hours before the MIC was recorded by visual inspection of growth.

### Multi-step resistance selection experiment

To determine if *S. aureus* would develop resistance to auranofin after repeated exposure, a multi-step resistance experiment was conducted, as described in previous reports^46,47^. The broth microdilution assay was used to determine the initial MIC for auranofin, clindamycin, and mupirocin, in triplicate, as described above. For each subsequent passage, an aliquot (5 µL) of bacterial culture, from wells below the MIC (sub-inhibitory concentration of drug) with turbidity similar to untreated control wells, was diluted 1:1000. The diluted culture was used to test the MIC of each test agent for the next passage. Plates containing bacteria and drugs were incubated at 37 °C for at least 18 hours before the MIC was determined by visual inspection. Bacteria were passaged for 14 days and resistance was characterized as a >4-fold increase in MIC relative to the initial MIC^18^.

### Postantibiotic effect of auranofin against staphylococci

The PAE for auranofin, clindamycin, and mupirocin was determined, in duplicate, using a method described in previous studies, with the following modifications^20,48^. Colonies of *S. aureus* ATCC 6538 (FDA 209), MRSA NRS123 (MRSA USA400), and MRSA NRS384 (MRSA USA300) were transferred to separate tubes containing TSB and incubated at 37 °C with agitation at 150 rpm until the incoula reached an OD_600_ ~ 1.0. Bacteria were diluted 1:1000 in TSB alone (negative control) or TSB containing 5 × MIC of auranofin, clindamycin, or mupirocin (each test agent evaluated in duplicates) and incubated for one hour at 37°C with agitation at 150 rpm. After treatment with each test agent, drugs were removed by diluting each tube 1:1000 in fresh TSB and incubating at 37 °C with agitation at 150 rpm for 12 hours. Samples were collected from each tube every hour, serially diluted in PBS, and plated onto TSA. TSA plates were incubated at 37 °C for at least 18 hours to determine viable CFU. The PAE was calculated using the following equation: *T* – *C*, where *T* is the time required for bacterial culture treated with drug to increase by one log_10_ (after washout of drug) and *C* is the time required for the negative control to increase by one log_10_.

### Infected pressure ulcer wound model in obese mice

This study was reviewed and approved by the Purdue University Animal Care and Use Committee and conducted in strict accordance with the National Institutes of Health Guide for the Care and Use of Laboratory Animals. Twelve-week-old female TALLYHO/JngJ mice (Jackson Laboratory, Bar Harbor, ME, USA) weighing on average 35 grams, were used for this study. Mice were housed in ventilated cages with access to water and food *ad libitum*. To induce infected pressure ulcers in mice, we developed a modified method to previously published reports^49–52^. One day prior to application of magnets, the fur along the dorsal region of mice was shaved and scrubbed with betadine solution. Thereafter, the dorsal skin just caudal to the scapulae was pinched and two 3,466 G, neodymium rare earth magnets (9.5 mm diameter × 3.2 mm thick) were placed on each side of the skin fold. Magnets were applied for ten ischemia-reperfusion cycles (two hours on, one hour off) to induce formation of moderate pressure ulcers. Mice received a subcutaneous injection of 0.03 mg/kg buprenorphine immediately before application of magnets and again every 12 hours later (during application of magnets) to minimize pain. Ulcers were infected one day after formation with 20 µL of 2.8 ×10^9^ CFU/mL MRSA NRS384 (USA300) and covered with Tegaderm fixed with Uro-Bond IV ostomy adhesive. The infection was allowed to proceed for two days before initiating treatment. On the first day of treatment, mice were randomly divided into groups of five mice. One group of mice was treated with clindamycin orally (30 mg/kg once daily). The infected pressure ulcers for the remaining groups were treated topically twice daily with either 1% auranofin, 2% auranofin, 2% mupirocin, or the vehicle used to prepare each topical treatment (petroleum jelly). Tegaderm was applied over the wounds and fixed with Uro-Bond IV adhesive after each treatment to prevent mice from biting, scratching, or grooming the area around the wounds. Mice were checked every four hours for signs of severe morbidity (e.g. hypothermia, inability to eat or drink, or significant weight loss (>20%)). All mice were humanely euthanized 12 hours after the last dose was administered via CO_2_ asphyxiation and the skin tissue around each ulcer was harvested aseptically. The ulcerated tissue was homogenized in sterile phosphate-buffered saline (PBS) using an Omni Tissue Homogenizer (TH115, Omni International, Kennesaw, GA, USA). To determine the bacterial load in the ulcers, the homogenate was serially diluted and plated on mannitol salt agar plates (to select for *S. aureus* colonies). Plates were incubated for at least 20 hours at 37 °C before bacterial colonies were enumerated. For the two auranofin treatment groups, 1 mL of homogenate was spread over four mannitol salt agar plates and plates were incubated for 48 hours at 37 °C to confirm the absence of MRSA colonies. Data are presented as log_10_ (MRSA CFU) in each wound.

### Statistical analysis

Data from the murine pressure ulcer experiment were analyzed using a one-way ANOVA with post-hoc Dunnet’s test for multiple comparisons (*P* < 0.05) using GraphPad Prism 8 (La Jolla, CA). To ensure the data was normally distributed, the data was subjected to the Kolmogorov-Smirnov test (*P* < 0.05).
